# Upper limb movements can be decoded from the time-domain of low-frequency EEG

**DOI:** 10.1371/journal.pone.0182578

**Published:** 2017-08-10

**Authors:** Patrick Ofner, Andreas Schwarz, Joana Pereira, Gernot R. Müller-Putz

**Affiliations:** Institute of Neural Engineering, BCI-Lab, Graz University of Technology, Graz, Austria; Shanghai Jiao Tong University, CHINA

## Abstract

How neural correlates of movements are represented in the human brain is of ongoing interest and has been researched with invasive and non-invasive methods. In this study, we analyzed the encoding of single upper limb movements in the time-domain of low-frequency electroencephalography (EEG) signals. Fifteen healthy subjects executed and imagined six different sustained upper limb movements. We classified these six movements and a rest class and obtained significant average classification accuracies of 55% (movement vs movement) and 87% (movement vs rest) for executed movements, and 27% and 73%, respectively, for imagined movements. Furthermore, we analyzed the classifier patterns in the source space and located the brain areas conveying discriminative movement information. The classifier patterns indicate that mainly premotor areas, primary motor cortex, somatosensory cortex and posterior parietal cortex convey discriminative movement information. The decoding of single upper limb movements is specially interesting in the context of a more natural non-invasive control of e.g., a motor neuroprosthesis or a robotic arm in highly motor disabled persons.

## Introduction

Understanding how the human brain encodes movements is essential for the development of an intuitive and natural control of a motor neuroprosthesis or a robotic arm. Neuroprostheses based on functional electrical stimulation (FES) [1] can be already used to restore movement function of spinal cord injured (SCI) persons [2]. These neuroprostheses often rely on a shoulder joystick as a control signal, and end users with SCI need to learn to control movements, such as grasping, with contralateral shoulder movements. However, this control would have a more natural feel for the end user if the movement intention is decoded with a brain-computer interface (BCI), and subsequently translated into a control signal for a neuroprosthesis or robotic arm. It has been shown with tetraplegic human subjects that invasive BCIs allow the control of a robotic arm with up to 10 degrees of freedom (DoF) [3–6]. Invasive BCIs have a better signal-to-noise ratio than non-invasive BCIs, but require extensive surgery, and the suitability for long-term use is still unclear due to neural tissue response. Non-invasive BCIs based on electroencephalography (EEG) signals on the other hand do not require surgery and are easier to setup. They often rely on power modulations of sensorimotor rhythms (SMR) accompanying movement imagination (MI) (see also event-related (de)synchronization [7]) but other brain rhythms can also be exploited [8,9]. These power modulations can act as the control signals for a neuroprosthesis [2,10–12]. Using an SMR-based BCI, our group has already shown the restoration of the lateral grasp of a tetraplegic (C4/5 ASIA A) user with MI of both feet [12]. In a later study, we demonstrated the switching between different lateral grasp phases in a person with SCI (C5 ASIA A) with an SMR-based BCI and the Freehand system [10]. Recently, Rohm et al. and Kreilinger et al. [11,13] restored not only hand but also elbow functions of a tetraplegic end user (a review can be found in Rupp et al. [2]). However, SMR-based BCIs can usually only detect spatially well separated patterns in the EEG as elicited by, for example, right hand MI vs left hand MI, although recent research suggests more spatially specific detections [14,15]. Furthermore, SMR-based BCIs usually require repetitive MI of movements. This often requires BCI users to learn unnatural MI commands, such as using repetitive left hand MI to control right hand functions [16]. However, for a more natural control the imagined movement should be as close as possible to the actual neuroprosthesis movement. In this context, continuous decoding of movement trajectories from the time-domain of the EEG has been investigated. Bradberry et al. showed in an offline study the decoding of 3D hand velocities [17], later our group showed the decoding of 3D positions in a continuous movement task [18] and the decoding of imagined movement trajectories [19]. Furthermore, Agashe et al. decoded hand joint angular velocities [20], and also hand movement directions were decoded non-invasively [21]. The current state of the art allows decoding of movement trajectories and directions from EEG, however the low correlation with the real or intended movement prevents a reliable and accurate control.

Another possibility to make neuroprosthesis or robotic arm control more natural is to decode additional information about the type or quality of an imagined movement, which has been done in the time-domain as well as in the frequency-domain of EEG. Gu et al. found that the speed of imagined wrist movements is encoded in the time-domain in motor-related cortical potentials (MRCPs) [22–24], and Yuan et al. found such a relationship in the mu and beta rhythm with executed/imagined hand movements [25]. Jochumsen et al. [26] decoded from MRCPs movement force and speed during executed and imagined grasping movements in healthy persons, and attempted movements in stroke patients. Also MIs related to the same limb were classified based on EEG power modulations in the frequency domain: Edelman et al. [15] classified repetitive imagined hand flexion/extension and forearm supination/pronation, Yong and Menon showed the classification of repetitive imagined grasp and elbow movements [14]. Based on these findings, the natural control experience can be enhanced if, e.g. an imagined repetitive supination of the arm is used to control the supination of, e.g. a robotic arm. Furthermore, detecting different MIs related to the same limb increases the number of control possibilities compared to classical SMR-based BCIs, which often only detect left/right hand and foot MI. However, repetitive MIs are also not optimal since one usually does not execute repetitive hand/arm movements when manipulating objects. Of special interest are therefore sustained MIs, such as single supination. Vučković and Sepulveda showed the classification of sustained wrist extension/flexion and forearm pronation/supination MIs from the frequency-domain of the EEG in the delta and gamma band [27,28]. Gu et al. classified imagined wrist extension and wrist rotation based on power-modulations in the mu and beta band and the rebound rate of MRCPs but did not find any statistical difference in the rebound rate of MRCPs [23].

In this work we hypothesize that executed and imagined sustained movements from the same limb can be decoded from low-frequency time-domain signals (< 3 Hz). We applied a multiclass classification comprising of 6 movement classes: elbow flexion/extension, forearm supination/pronation, and hand open/close. Additionally, these movements were classified against a rest class. We measured 15 healthy subjects in two separate ME and MI sessions. To the best of our knowledge, this high number of different sustained movements of the same limb has not been studied before using low-frequency time-domain EEG signals. Furthermore, we show for the first time for EEG-based movement decoding the classifier patterns [29] in the source space, which allows the estimation of the brain regions exploited by the classifier. Generally, the purpose of this work is to get a better understanding if and how single sustained upper limb movements are encoded in the time-domain of low-frequency EEG signals.

## Methods

### Subjects

We recruited 15 healthy subjects aged between 22 and 40 years with a mean age of 27 years (standard deviation 5 years). Nine subjects were female, and all the subjects except s1 were right-handed. The subjects received payment for their participation. Written informed consent was obtained from all subjects, and the study was conducted in accordance with the protocol approved by the ethics committee of the Medical University of Graz (approval number 28–108 ex 15/16).

### Paradigm

**Fig 1:**
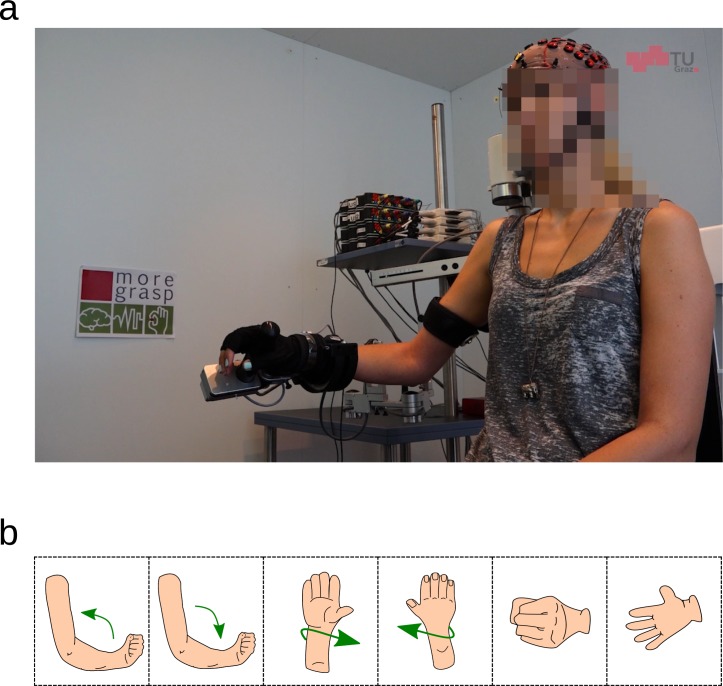
Experimental setup and movements. **a:** Subjects sat in a chair and executed/imagined movements according to cues presented on a computer screen in front of them. **b:** Subjects executed/imagined: elbow flexion, elbow extension, forearm supination, forearm pronation, hand close, and hand open.

Subjects sat on a chair and their right arm was fully supported by an exoskeleton with anti-gravity support (Hocoma, Switzerland) to avoid muscle fatigue, see Fig 1A (the individual in this figure has given written informed consent, as outlined in PLOS consent form, to publish these case details).

We measured each subject in two sessions on two different days, which were not separated by more than one week. In the first session the subjects performed ME, and MI in the second session. The subjects performed six movement types which were the same in both sessions and comprised of elbow flexion/extension, forearm supination/pronation and hand open/close; all with the right upper limb (see Fig 1B). All movements started at a neutral position: the hand half open, the lower arm extended to 120 degree and in a neutral rotation, i.e. thumb on the inner side. Additionally to the movement classes, a rest class was recorded in which subjects were instructed to avoid any movement and to stay in the starting position. In the ME session, we instructed subjects to execute sustained movements. In the MI session, we asked subjects to perform kinesthetic MI [30] of the movements done in the ME session (subjects performed one ME run immediately before the MI session to support kinesthetic MI).

**Fig 2:**
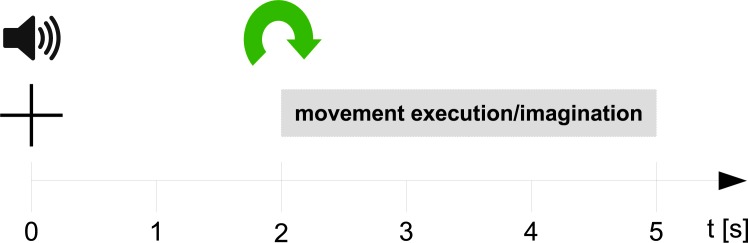
Trial sequence. At second 0, a cross appeared together with a beep sound; at second 2, the cue was presented and subjects executed/imagined a sustained movement or avoided any movement, respectively. After the trial, a break with a random duration of 2 s to 3 s followed.

The paradigm was trial-based and cues were displayed on a computer screen in front of the subjects, Fig 2 shows the sequence of the paradigm. At second 0, a beep sounded and a cross popped up on the computer screen (subjects were instructed to fixate their gaze on the cross). Afterwards, at second 2, a cue was presented on the computer screen, indicating the required task (one out of six movements or rest) to the subjects. At the end of the trial, subjects moved back to the starting position. In every session, we recorded 10 runs with 42 trials per run. We presented 6 movement classes and a rest class and recorded 60 trials per class in a session.

### Recording

The EEG was measured from 61 channels covering frontal, central, parietal and temporal areas using active electrodes and four 16-channel amplifiers (g.tec medical engineering GmbH, Austria). Reference was placed on the right mastoid, ground on AFz. We used an 8^th^ order Chebyshev bandpass filter from 0.01 Hz to 200 Hz and sampled with 512 Hz. Power line interference was suppressed with a notch filter at 50 Hz. In addition we measured the arm joint angles for the exoskeleton using customized software and finger positions with a 5DT Data Glove (5DT, USA) for determining movement onsets. Prior to each session, we measured the electrode positions with a CMS 20 EP system (Zebris Medical GmbH, Germany). The individual electrode positions were used for source imaging.

### Movement onset detection

To detect movement onsets in ME sessions we used sensor data from the exoskeleton and the data glove. The elbow and wrist sensors (exoskeleton) were used to detect elbow flexion/extension and forearm pronation/supination onsets, respectively. For opening/closing onsets we performed a principal component analysis on the data glove sensor data and used only the first principal component for further processing. A movement was detected when the absolute difference between the sensor data and the preceding time average (from -1 s to -0.5 s) crossed a threshold. Thresholds were chosen dependent on each sensor to ensure timely detection of movement onsets and to minimize false positive detections (typically, movements were detected not more than 80 ms later than a human expert would detect them when visually inspecting the sensor data). In order to account for systematic detection time differences between the classes (e.g. different sensor thresholds and different inertiae of limb parts), we time-shifted the mean value of the detection times of each class toward the mean value of all classes. Thus, on average the movement onsets (wrt. to the cue) of the movement classes were all the same. For the classes without overt movements (i.e., the rest class and the MI classes), we assumed a virtual movement onset. This virtual movement onset was individually calculated for each subject as the average movement onset of the movement classes. In this manner, all classes were still comparable.

### Preprocessing

We used EEGLAB to detect and remove noisy channels (1.4 channels per subject on average) based on the joint probability of each channel. We downsampled the data to 256 Hz to save computation time. Thereafter we marked artefacts by band-pass filtering (0.3 Hz—70 Hz, 4^th^ order zero-phase Butterworth filter) the data and using EEGLAB[31] to find (1) values above/below thresholds of -200 μV and 200 μV, respectively, (2) trials with abnormal joint probabilities, and (3) trials with abnormal kurtosis. The methods (2) and (3) used as threshold 5 times the standard deviation of their statistic to detect artefact contaminated trials. The artefact contaminated trials were only marked for removal but not yet removed. Afterwards, we filtered the original (unfiltered) 256 Hz EEG data with a zero-phase 4^th^ order Butterworth filter between 0.3 Hz and 3 Hz and re-referenced the data to a common average reference. Subsequently, we discarded the trials previously marked as artefact contaminated.

### Classification

The preprocessed signals were classified with a shrinkage regularized linear discriminant analysis (sLDA) classifier [32,33] which was embedded in the discriminative spatial pattern (DSP) [29] framework described in the next section.

We conducted two types of classifications: first, we classified all 6 movement classes against each other. Second, we aggregated all movement classes into one class and classified it against the rest class. We refer to these classification types as mov-vs-mov and mov-vs-rest classifications, respectively. In the mov-vs-rest classification we randomly removed trials from the aggregated movement class to ensure equal trial numbers in both classes. As mov-vs-mov was a multiclass classification comprising of 6 classes, we applied an 1-vs-1 classification strategy yielding 15 binary classifiers. To validate the classification we employed a 10x10-fold cross-validation.

We employed two classification approaches using EEG data from: (1) single time points and (2) time windows with different lengths (0.2–1 s). Single time point classification gives a higher time resolution of the accuracy course and is more suitable to analyze the information distribution over time. Furthermore, the corresponding classifier patterns can be readily obtained with the DSP method described in the next section. The time window based classification, on the other hand, is expected to increase the classification accuracy. Because every method has its benefits, we analyzed both approaches in this work and refer to them as “single time point” and “time window” based classifications.

### Classifier patterns

We calculated the classifier pattern based on the discriminative spatial pattern (DSP) method [29]. This method allows the calculation of an (s)LDA classifier and the corresponding patterns simultaneously. An LDA can be formulated as an optimization problem of Fisher's’ criterion and consecutively as a generalized eigenvalue problem. When this generalized eigenvalue problem is solved for the eigenvector corresponding to the largest eigenvalue one obtains the LDA weight vector. DSP also solves this generalized eigenvalue problem for the remaining eigenvectors and one obtains a weight matrix. This weight matrix can then be inverted to obtain the pattern.

Let x(t) be a vector of the EEG channels at time t with dimension [channels x 1], w_t_ the computed LDA weight vector at time t with dimension [channels x 1], and the scalar y(t) the projection of the original EEG channels to the LDA space. Then the LDA can be formulated as

$${x(t)}^{T}\cdot w_{t}=y(t)$$ (1)

and w_t_ corresponds to the eigenvector with the largest eigenvalue. With DSP we get a weight matrix instead where the first column (when sorted by the eigenvalue) corresponds to the LDA solution:

$${x(t)}^{T}\cdot W_{t}(:,1)=y(t)$$ (2)

This weight matrix can be inverted to obtain the pattern a_t_ corresponding to the LDA weights:

$$A_{t}=W_{t}^{-1}$$ (3)

$$a_{t}=A_{t}(1,:)$$ (4)

In fact, we obtained an sLDA weight vector because we calculated the within-class scatter matrix (a factor in the Fisher's criterion) using shrinkage regularization. We calculated the patterns for every time step in the time window from -0.4 s to 0.4 s relative to the movement onset (indicated by the subscript t).

In general terms, a pattern explains how a source, e.g. a specific brain area or independent component, is projected on the channels. Noteworthy, “source” can refer to two different concepts: first, the sources constituting a classifier (manifesting as a pattern) in channel space (i.e. scalp potential distribution), second, the brain sources found with source imaging methods, i.e. voxels. This section refers solely to patterns and the next section shows how source imaging was applied to transform this pattern to the source (voxel) space. Each element in a pattern vector shows with what impact a source is projected on the associated channel. It is important to bear in mind that a pattern itself does not have any physical representation, i.e. it has no physical unit. However, a common physical unit would be a necessity when averaging and interpreting patterns. If we multiply (scale) a source with its pattern, we get the projection to the channel space in the same physical unit as the source, e.g. if the source corresponds to Volt, the resulting scaled pattern corresponds to Volt too. In the case of LDA, however, we do not have a single source but two classes in the channel space projected into an one dimensional LDA space. Thus, we are interested in the distance between the two classes in the LDA space. In our scaling approach we use the distance between the two class means in the LDA space as a scaling factor for a_t_. Let μ_0,t_ and μ_1,t_ be vectors with dimension [channels x 1] representing the class means of the two classes in the channel space, then the scaled pattern can be calculated as:

$$a_{scaled,t}=(\mu_{0,t}^{T}\cdot w_{t}-\mu_{1,t}^{T}\cdot w_{t})\cdot a_{t}$$ (5)

With this scaling we get a pattern which has the same physical unit as the original channel space. The pattern shows the differences of the class means in the original space as exploited by the LDA classifier. We then transformed this pattern from the channel space into the source space using standardized low-resolution brain electromagnetic tomography (sLORETA) [34], see the next section for more details.

**Fig 3:**
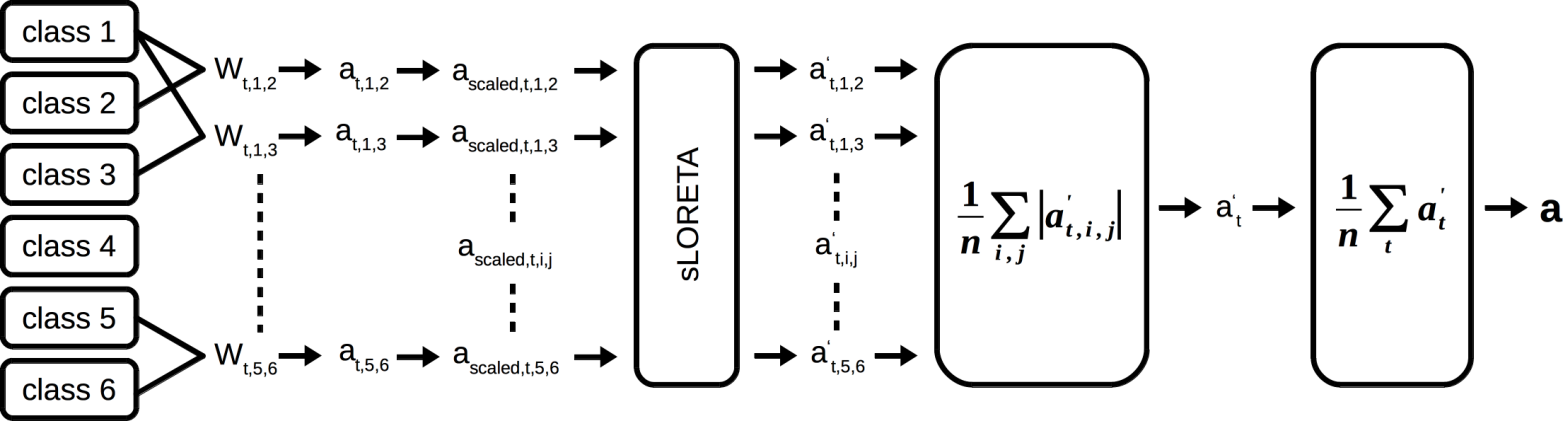
Calculation of a mov-vs-mov pattern. Patterns are calculated from each 1-vs-1 classifier; subsequently scaled and transformed into the source space; we then calculated the absolute value and averaged over patterns. Finally, we averaged over non-overlapping time segments. The same processing pipeline applies to the mov-vs-rest pattern.

As we applied an 1-vs-1 classification strategy, we obtained several binary classifiers and therefore also several patterns (e.g. a supination vs pronation pattern). To obtain the final classifier patterns we grouped the patterns according to the two classification types: movement vs movement patterns (mov-vs-mov) and movement vs rest patterns (mov-vs-rest). Patterns belonging to a group were averaged using their absolute values. We took the absolute values because a pattern expresses the difference between two classes and its signs depend on the order of the classes and should therefore not be considered. Finally, we averaged the patterns over non-overlapping 100 ms time segments located between -0.4 s and 0.4 s relative to the movement onset, i.e. yielding 9 patterns per classification type for each session and subject. Additionally, we time averaged over the whole -0.4 s to 0.4 s period. Fig 3 summarizes the procedure.

### Source space

EEG source imaging methods allow to infer from the EEG (i.e. scalp potential distribution) the underlying sources in the brain. The EEG signals are attributed to the “channel space”, whereas the inferred brain sources are attributed to the “source space” and are often estimated (normalized) current densities [35].

We transformed the LDA patterns (obtained from single EEG time-points) from the channel space into the source space to increase the spatial resolution of the patterns obtained. For this purpose, we used the software Brainstorm [36]. A desirable property of scaled LDA patterns compared to LDA weights is that they correspond to measured scalp potentials and can be subjected to source imaging methods similar as EEG channels. Boundary element head models were calculated based on subject individual electrode positions and the ICBM152 template head model (ICBM152 is a head model based on a non-linear average of 152 subjects). We estimated the full noise covariance matrices based on the EEG data from the period 0.5 to 2 s after trial start and applied shrinkage regularization [37]. Finally, we computed 15002 brain sources, i.e. voxels, with sLORETA [34] (the dipole orientations were unconstrained).

### Classifier pattern statistics

Group level statistics was done by nonparametric permutation testing [38,39] of the classifier patterns in the source space. The statistical testing was done separately for each ME/MI and mov-vs-mov/mov-vs-rest pattern. Beside the actual classifier patterns, we calculated random classifier patterns by shuffling class labels once for each subject. As a test statistic, we used the difference between the actual classifier patterns and the random classifier patterns averaged over all subjects. We obtained the permutation distribution of the test statistic by enumerating all 2^15^ = 32768 actual/random classifier pattern combinations. For that, we used the maximum of the voxels in each enumeration step to account for multiple comparisons (in case of 100 ms time segments, we used the maximum of the whole -0.4 s to 0.4 s period). We then established a threshold corresponding to α = 0.05. All voxels with a test statistic exceeding the threshold were considered significant.

## Results

### Classification accuracies

**Fig 4:**
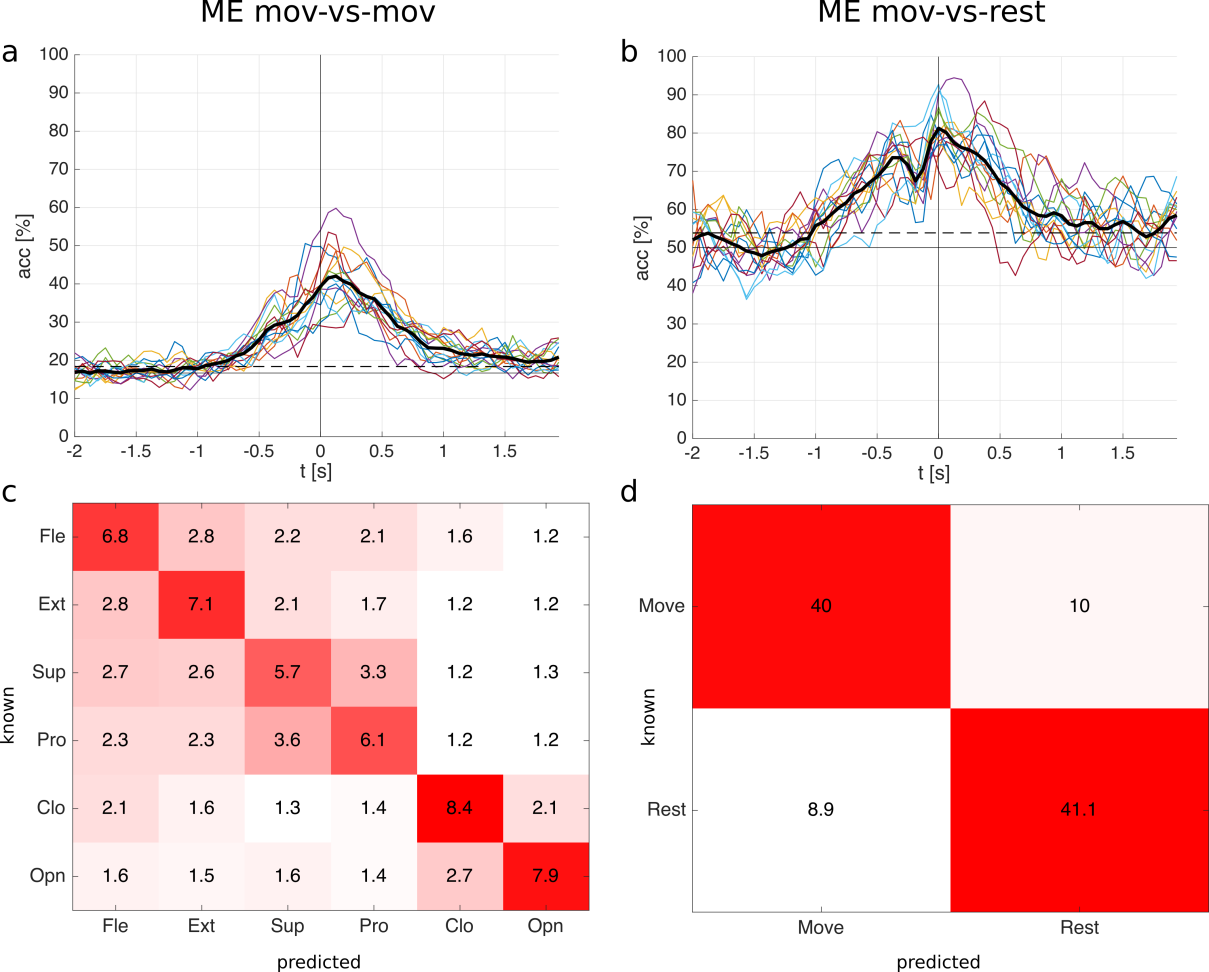
ME classification results for the single time point classification. **a:** mov-vs-mov classification accuracies of all 15 subjects and the average (thick black line). Time point 0 s corresponds to the movement onset. **b:** mov-vs-rest classification accuracies. The horizontal solid line in a and b is the chance level; the horizontal dashed line is the significance level for the average. **c:** mov-vs-mov confusion matrix (occurrences sum to 100%) with classes elbow flexion (Fle), elbow extension (Ext), forearm supination (sup), forearm pronation (pro), hand close (Clo), and hand open (Opn). **d:** mov-vs-rest confusion matrix. Confusion matrices were calculated at the time point with the highest average classification accuracy (mov-vs-mov: 0.13 s; mov-vs-rest: 0.0 s).

Single time point classification. The ME classification accuracies are shown in Fig 4A (mov-vs-mov) and Fig 4B (mov-vs-rest). The mov-vs-mov average classification accuracy over all subjects reached a maximum of 42% (9% standard deviation) at 0.13 s after movement onset and the mov-vs-rest average classification accuracy reached a maximum of 81% (7% standard deviation) at movement onset (0.0 s). Accuracies were calculated from -2 s to 2 s relative to the movement onset with a time resolution of 1/16 s. Classification accuracies are statistically significant above 24% (mov-vs-mov) and 65% (mov-vs-rest) for a single subject, and above 18% (mov-vs-mov) and 54% (mov-vs-rest) for the average (α = 0.05, adjusted wald interval [40,41], Bonferroni corrected for the length of the analyzed time window). We calculated the significance levels based on the average number of trials available after artefact removal. In mov-vs-mov and mov-vs-rest all subjects reached a significant classification accuracy, see Table 1 which shows the individual maximum classification accuracies. The mov-vs-mov averaged classification accuracy becomes significant at -0.94 s and stays significant until the end of the analyzed time window (2 s); the mov-vs-rest averaged classification accuracy is significant between -1.0 s and 1.69 s, see Fig 4A and 4B.

**Table 1:** Maximum ME classification accuracies for the single time point classification.

ME	s1	s2	s3	s4	s5	s6	s7	s8	s9	s10	s11	s12	s13	s14	s15	avg
mov-vs-mov [%]	**51**	**51**	**39**	**60**	**36**	**38**	**36**	**40**	**49**	**50**	**39**	**43**	**42**	**54**	**40**	44 ± 7
mov-vs-rest [%]	**85**	**81**	**83**	**94**	**87**	**93**	**78**	**81**	**83**	**80**	**79**	**86**	**91**	**88**	**81**	85 ± 5

Included is the average and standard deviation over subjects. Significant classification accuracies are bold.

Confusion matrices are shown in Fig 4C (mov-vs-mov) and Fig 4D (mov-vs-rest). They correspond to the timepoints when the average classification accuracies reached a maximum. The confusion matrices show relative numbers, i.e. the occurrences sum up to 100%. If a movement was wrongly predicted, it was often predicted as a movement involving the same joints, see Fig 4C. In other words, movements involving different joints (e.g. open vs pronation) are better distinguishable than movements involving the same joints (e.g. open vs close).

**Fig 5:**
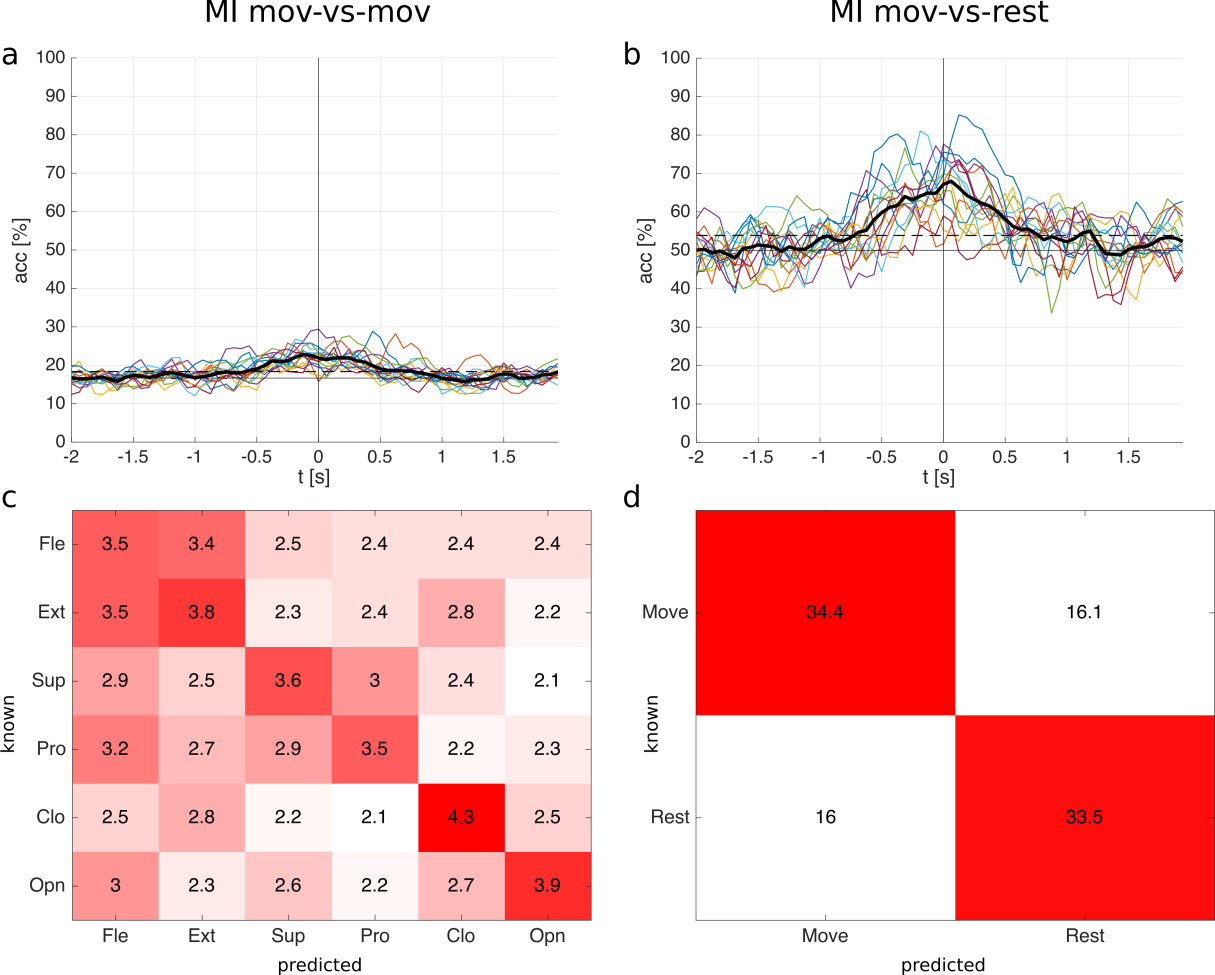
MI classification results for the single time point classification. **a:** mov-vs-mov classification accuracies of all 15 subjects and the average (thick black line). Time point 0 s corresponds to the movement onset. **b:** mov-vs-rest classification accuracies. The horizontal solid line in a and b is the chance level; the horizontal dashed line is the significance level for the average. **c:** mov-vs-mov confusion matrix (occurrences sum to 100%). **d:** mov-vs-rest confusion matrix. Confusion matrices were calculated at the time point with the highest average classification accuracy (mov-vs-mov: -0.13 s; mov-vs-rest: 0.06 s).

Fig 5 shows the MI classification accuracies. The mov-vs-mov average classification accuracy over all subjects reached a maximum of 23% (3% standard deviation) at -0.13 s; the mov-vs-rest average classification accuracy reached a maximum of 68% (8% standard deviation) at 0.06 s. Accuracies are significant above 24% (mov-vs-mov) and 65% (mov-vs-rest) for a single subject, and above 18% (mov-vs-mov) and 54% (mov-vs-rest) for the average (α = 0.05, adjusted wald interval, Bonferroni corrected for the length of the analyzed time window). Ten subjects reached a significant classification accuracy in mov-vs-mov and 15 subjects in mov-vs-rest, see Table 2. The mov-vs-mov average classification becomes significant between -0.56 s and 0.81 s; the mov-vs-rest average classification is significant between -0.69 s and 0.81 s, see Fig 5A and 5B.

**Table 2:** Maximum MI classification accuracies for the single time point classification.

MI	s1	s2	s3	s4	s5	s6	s7	s8	s9	s10	s11	s12	s13	s14	s15	avg
mov-vs-mov [%]	**29**	23	23	**29**	**24**	**24**	**24**	**26**	**28**	23	22	**28**	**27**	**25**	23	25 ± 2
mov-vs-rest [%]	**71**	**72**	**68**	**78**	**77**	**81**	**66**	**85**	**68**	**66**	**77**	**66**	**74**	**73**	**76**	73 ± 6

Included is the average and standard deviation over subjects. Significant classification accuracies are bold.

The averaged maximum mov-vs-mov accuracies are 1.8 times higher for ME than for MI, the averaged maximum mov-vs-rest accuracies are 1.2 times higher for ME than for MI (cf. Table 1 and Table 2). The ME and MI accuracies are significantly different for mov-vs-mov and mov-vs-rest (p < 5⋅10^−4^, two-sided Wilcoxon signed rank test).

MI confusion matrices are shown in Fig 5C (mov-vs-mov) and Fig 5D (mov-vs-rest). They qualitatively show similar patterns as in ME, i.e. MI involving different joint are better discriminable than MI involving same joints.

**Fig 6:**
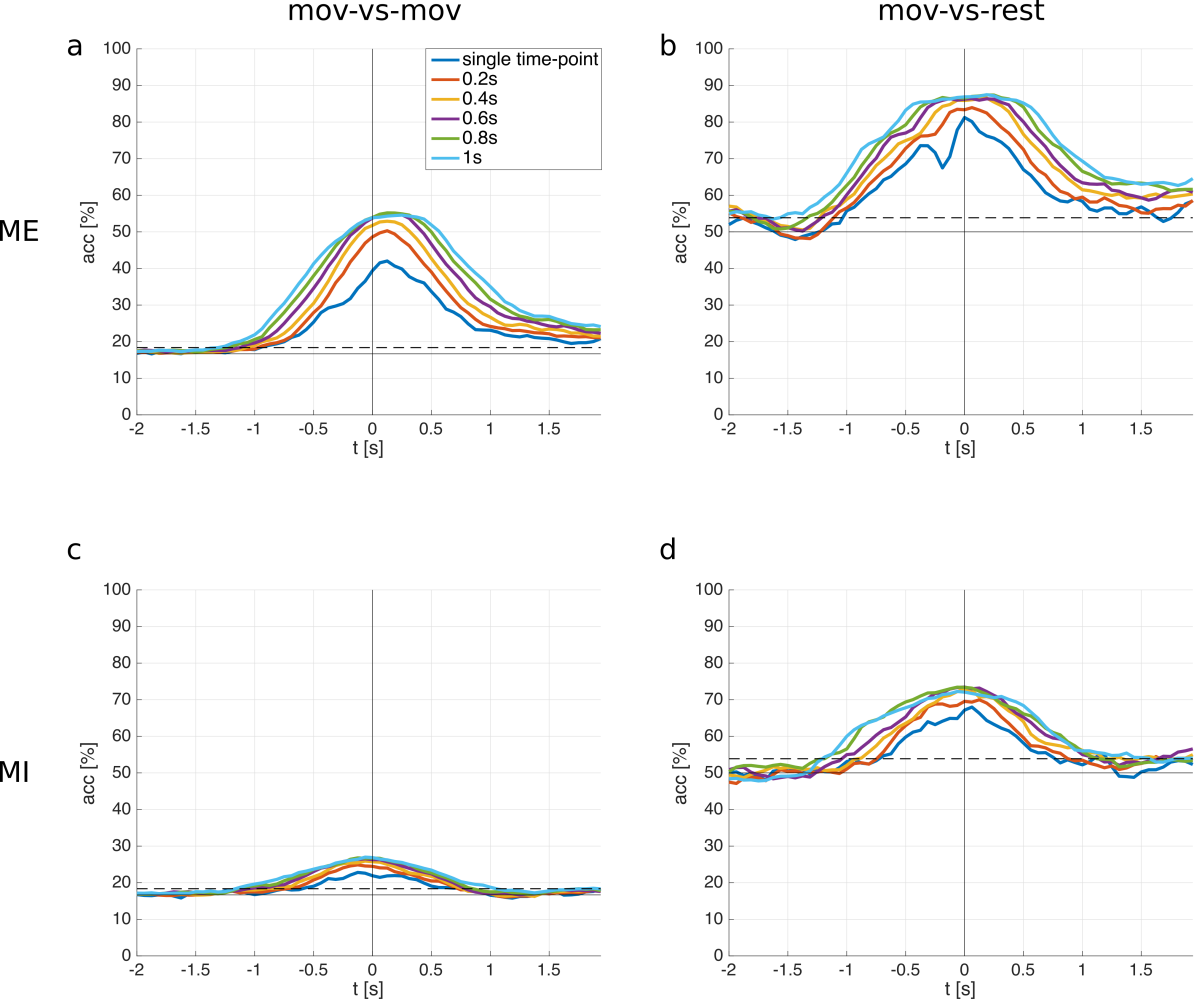
Classification accuracies for different window lengths. Time point 0 s corresponds to the movement onset. The horizontal solid lines are the chance level; the horizontal dashed lines are the significance levels. **a:** subject averaged ME mov-vs-mov classification accuracies. **b:** subject averaged ME mov-vs-rest classification accuracies. **c:** subject averaged MI mov-vs-mov classification accuracies. **d:** subject averaged MI mov-vs-rest classification accuracies.

Time window classification. Beside classifying on single time points, we also classified time windows of the EEG. The analyzed time windows ranged from 200 ms to 1 s, and features were taken in 100 ms time intervals within these time windows (see Table 3). Fig 6 shows the subjects' averaged ME/MI classification accuracies for the different window lengths as well as single time-point classification (relative to the movement onset) for comparison. The maximum averaged classification accuracies, the respective time points and standard deviations can be read from Table 4 (ME) and Table 5 (MI), respectively. Accuracies are significant above 18% (ME/MI mov-vs-mov) and 54% (ME/MI mov-vs-rest) (α = 0.05, adjusted wald interval, Bonferroni corrected for the length of the analyzed time window).

**Table 3:** Time windows used for classification.

window length [s]	number of time points fed into the classifier
0 (single time point)	1
0.2	3
0.4	5
0.6	7
0.8	9
1	11

**Table 4:** ME classification accuracies for different window lengths.

ME	window length					
	0 s	0.2 s	0.4 s	0.6 s	0.8 s	1 s
**mov-vs-mov**						
max acc [%]	42	50	53	55	55	55
std dev [%]	9	9	10	10	9	9
time [s]	0.13	0.13	0.13	0.13	0.13	0.25
**mov-vs-rest**						
max acc [%]	81	84	87	86	87	87
std dev [%]	7	6	6	4	6	4
time [s]	0.0	0.06	0.19	-0.13	0.19	0.19

Included is the maximum of the average classification accuracy and the respective standard deviation and time point.

**Table 5:** MI classification accuracies for different window lengths.

MI	window length					
	0 s	0.2 s	0.4 s	0.6 s	0.8 s	1 s
**mov-vs-mov**						
max acc [%]	23	25	26	27	27	27
std dev [%]	2	3	4	3	4	3
time [s]	-0.13	-0.13	-0.06	-0.06	-0.13	-0.06
**mov-vs-rest**						
max acc [%]	68	70	73	73	73	72
std dev [%]	8	8	5	7	7	8
time [s]	0.06	0.13	0.0	0.0	-0.06	-0.06

Included is the maximum of the average classification accuracy and the respective standard deviation and time point.

**Fig 7:**
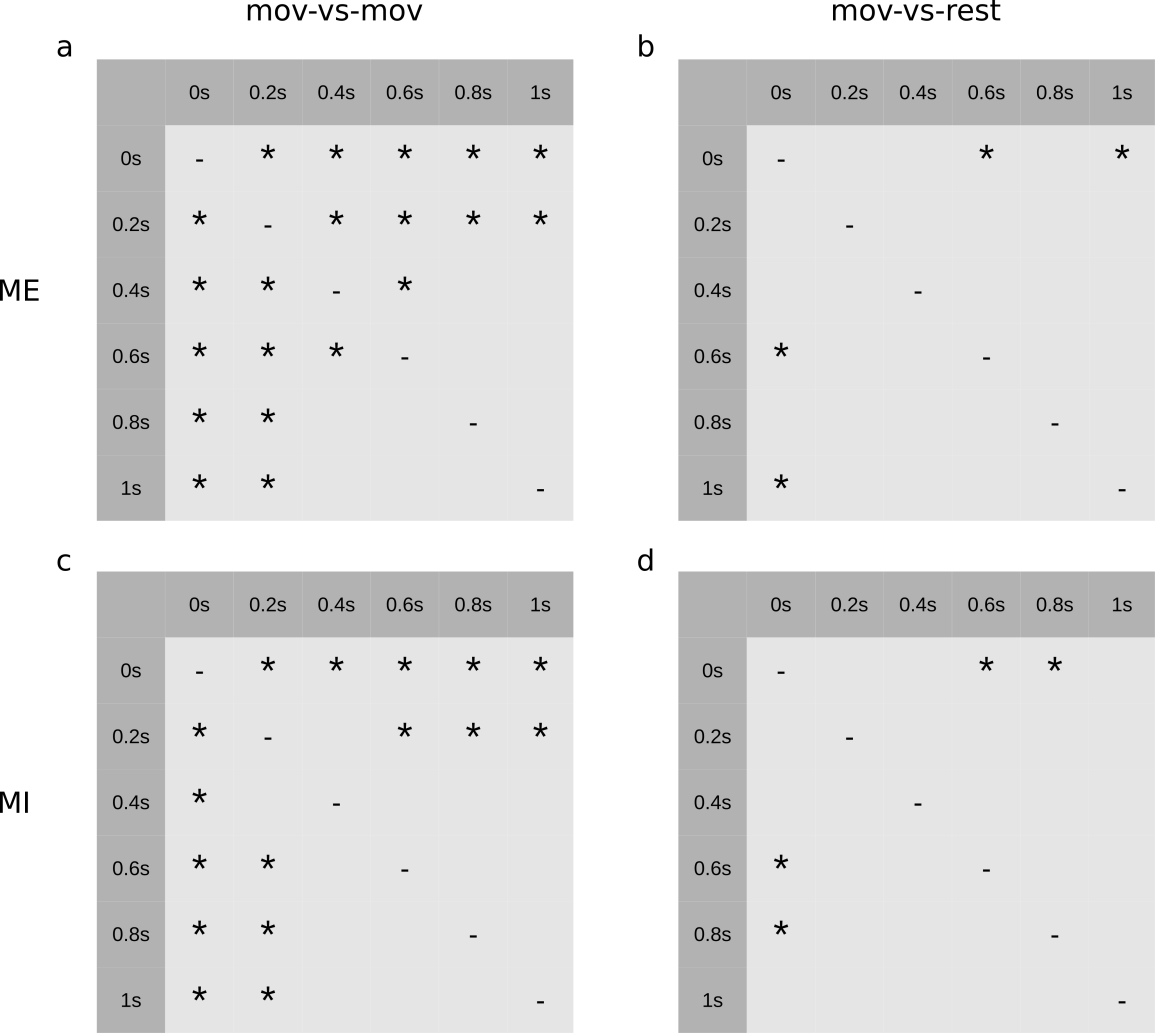
Post hoc tests with Dunn & Šidák's method between window lengths. A star indicates a statistically significant difference (p < 0.05) **a:** ME mov-vs-mov **b:** ME mov-vs-rest **c:** MI mov-vs-mov **d:** MI mov-vs-rest.

A one-way repeated measures ANOVA was conducted to compare the effect of the window length on the classification accuracy (at the time point of maximum average classification accuracy). There was a statistically significant effect for the window length for ME mov-vs-mov [F(5,70) = 59.2, p_GG_ = 7.0e-11], ME mov-vs-rest [F(5,70) = 7.1, p_GG_ = 0.002], MI mov-vs-mov [F(5,70) = 21.6, p = 5.0e-13], and MI mov-vs-rest [F(5,70) = 3.5, p_GG_ = 0.02]. Mauchly's test indicated that the sphericity assumption had been violated for ME mov-vs-mov, ME mov-vs-rest and MI mov-vs-rest (p < 0.05), and a Greenhouse-Geisser correction was applied in these cases. Post hoc tests with Dunn & Šidák's method were performed between groups and results are shown in Fig 7.

### Motor-related cortical potentials

**Fig 8:**
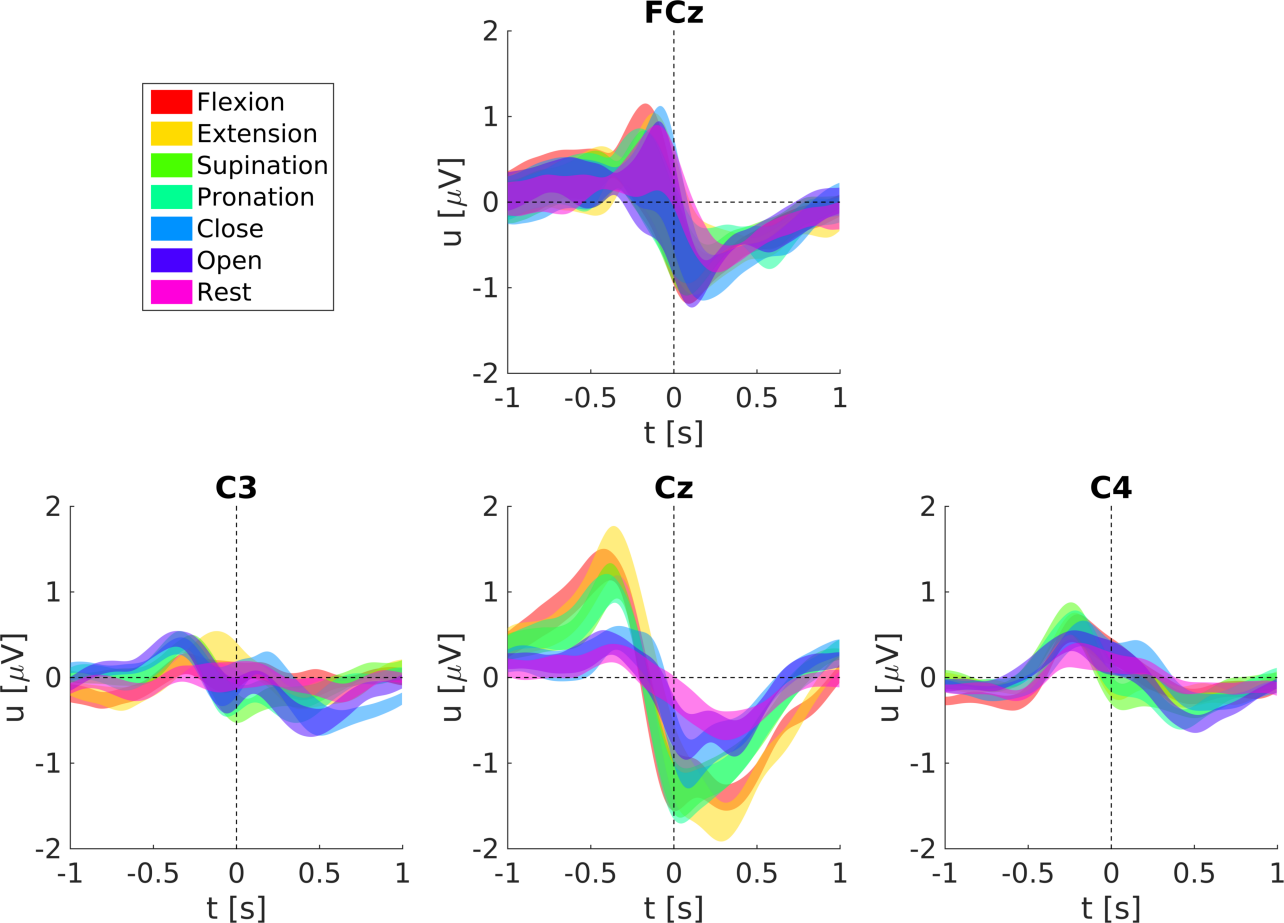
Grand-average MRCPs and respective standard errors during ME.

**Fig 9:**
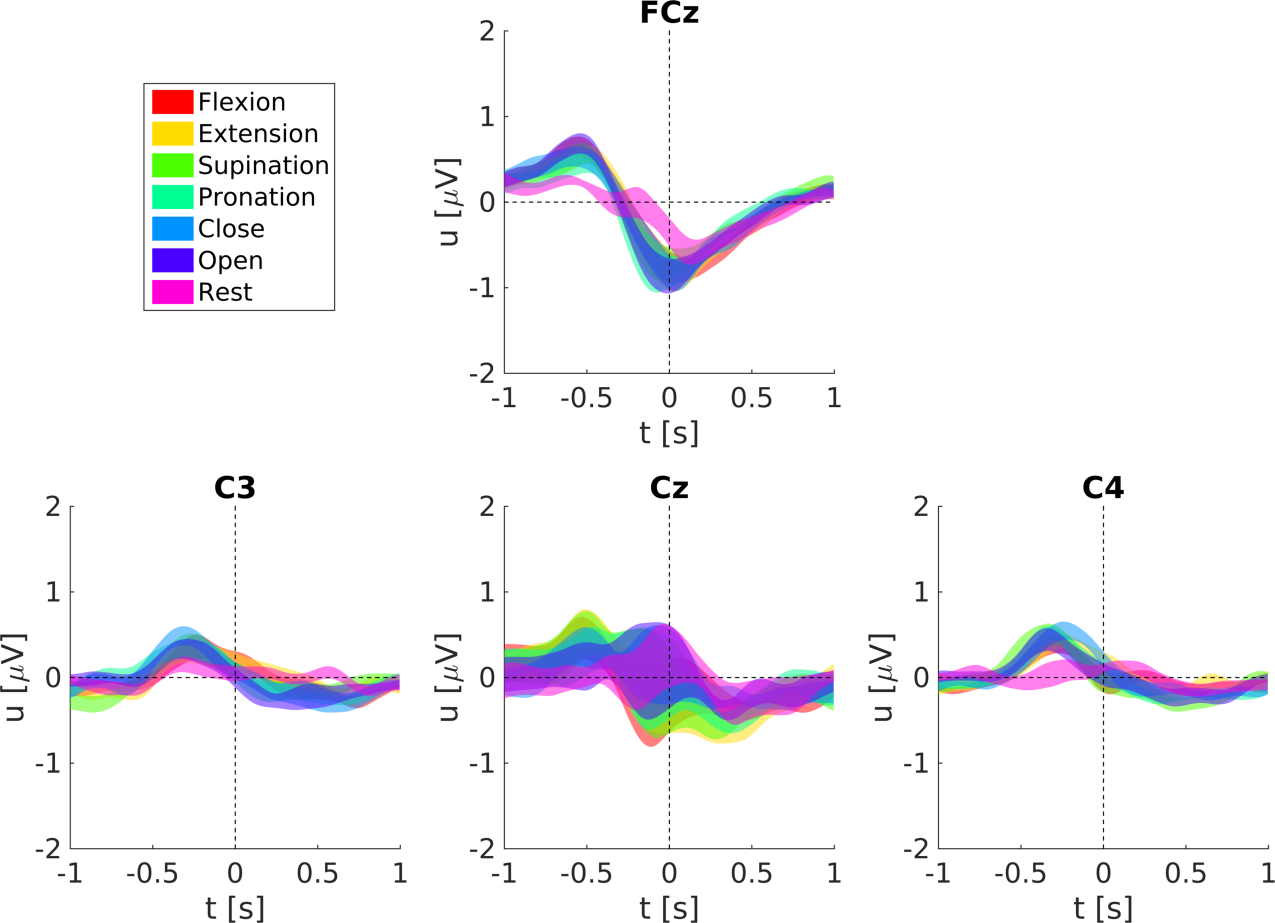
Grand-average MRCPs and respective standard errors during MI.

The grand-average MRCPs for all movements and the rest condition are shown in Fig 8 (ME) and Fig 9 (MI). MRCPs are aligned to the movement onset for ME and the virtual movement onset for MI, respectively. We show the grand-average MRCPs for channels FCz, C3, Cz, and C4, here Laplace filtered to increase the spatial resolution, the preprocessing was otherwise the same as for the classification. Laplace filtering was done by subtracting the mean voltage of the four surrounding orthogonal electrodes from the center electrode [42]. Generally, ME MRCPs are more pronounced than MI MRCPs (especially on Cz), and the rest condition shows smaller but otherwise similar shaped responses as the movements. The MRCPs show the largest response on Cz (ME) and on FCz (MI), respectively.

**Fig 10:**
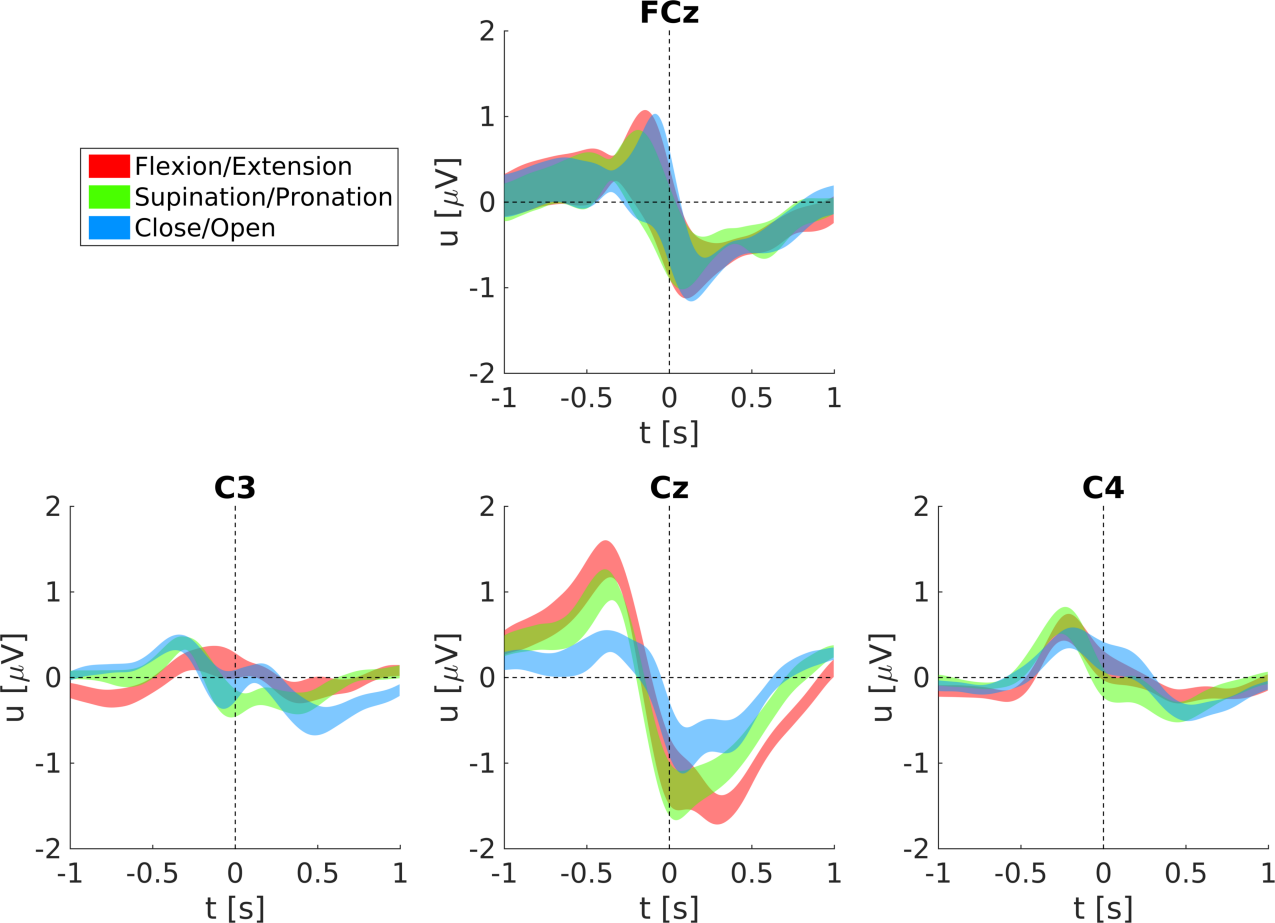
Grand-average ME MRCPs grouped with respect to their joint movements and respective standard errors. Shown are the averages of elbow extension/flexion, forearm supination/pronation and hand opening/closing.

Fig 10 shows the ME MRCPs averaged over all subjects with respect to their joint movements. MRCPs on Cz for forearm supination/pronation and elbow flexion/extension are more pronounced than for hand close/open. Elbow and forearm pronation/supination movements have similar MRCPs prior to movement onset and show differences in the latency of their negative peak (around 50 ms and 300 ms, respectively). Also differences in the MRCPs of movements belonging to the same joint are observable (see S1 Fig). The negative peak at Cz in hand opening is 0.3 μV larger than in hand closing. Almost no differences in latency or amplitude can be found between forearm pronation and supination. Elbow flexion leads to earlier MRCPs at Cz (around 60 ms) and weaker MRCPs (about 0.3 μV) than elbow extension. Such a detailed and fair comparison of the MI MRCPs between conditions is not reasonable, since the real imagined movement onset cannot be given.

### Classifier patterns

**Fig 11:**
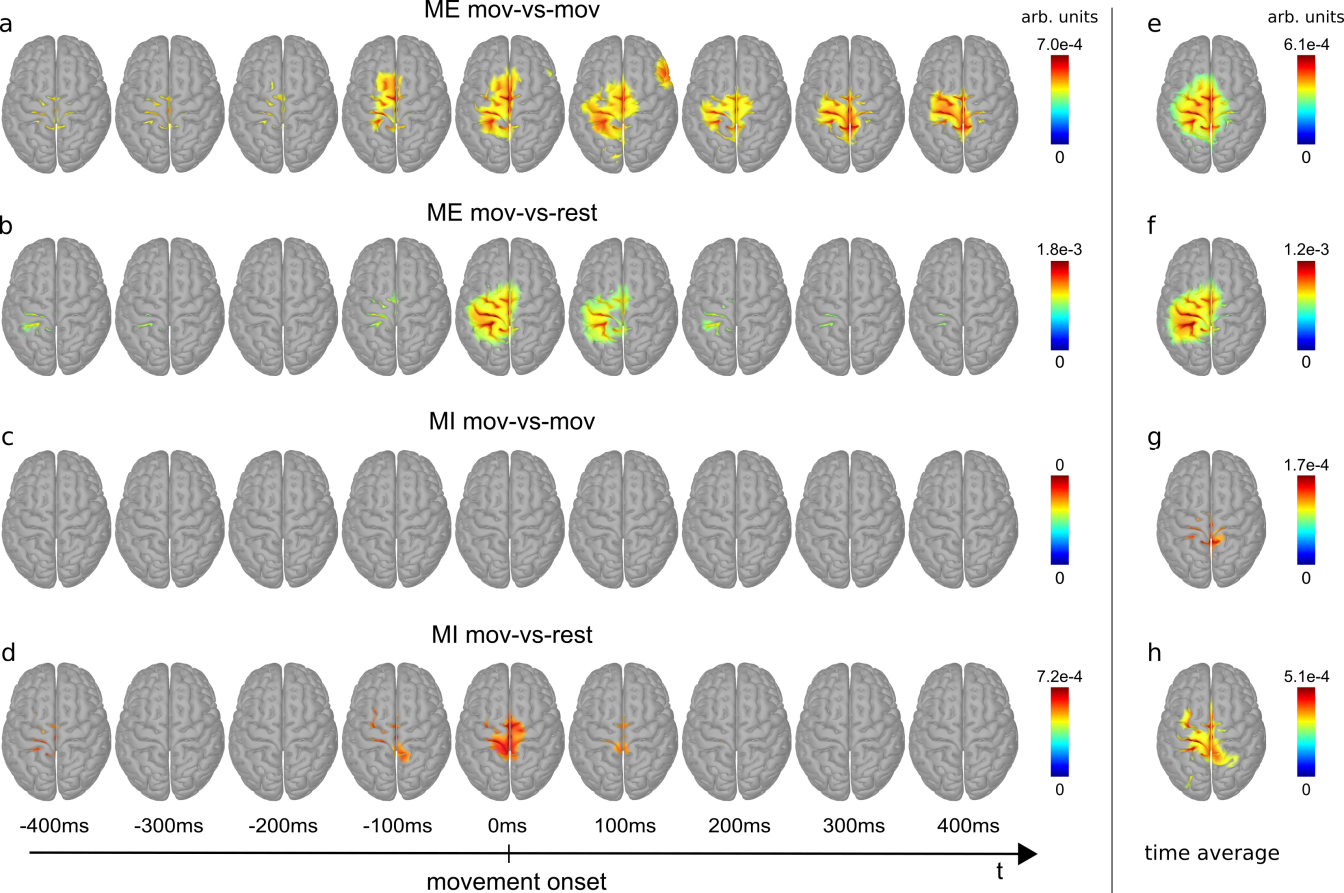
Classifier patterns. Shown are patterns between -0.4 s and 0.4 s relative to movement onset (**a**-**d**) and averaged over this time period (**e**-**h**). **a** and **e:** mov-vs-mov patterns during ME. **b** and **f:** mov-vs-rest patterns during ME. **c** and **g:** mov-vs-mov patterns during MI. **d** and **h:** mov-vs-rest patterns during MI. Only significant voxels are colored. Blue corresponds to zero, red to the maximum value.

We calculated 9 classifier patterns per subject, per classification type (mov-vs-mov and mov-vs-rest), and per movement condition (ME, MI), ranging from -0.4 s to 0.4 s relative to movement onset. Additionally, we calculated classifier patterns averaged over this time period. We subjected these patterns to statistical analysis, as described in the Methods section, and show them in Fig 11. The figure shows the group averages of the differences between classifier patterns and random classifier patterns (i.e. reference patterns) and only significant voxels are colored.

Immediately before movement onset (around -100 ms), the ME mov-vs-mov patterns (see Fig 11A) are prominent on premotor areas (PM). Subsequently (0–100 ms), patterns intensify on the contralateral primary motor (M1), contralateral somatosensory cortex (S1) and the posterior parietal cortex (PPC). After 300 ms, patterns remain on M1 and S1. Patterns are shortly observable on an ipsilateral temporal area (100 ms). In the ME mov-vs-rest condition (see Fig 11B) patterns appear at movement onset (0 ms) contralaterally on PM, M1, S1 and PPC. The pattern on PM vanishes 100 ms after movement onset and the remaining patterns vanish almost entirely 200 ms after movement onset. S1 Video and S2 Video show the progression of the mov-vs-mov and mov-vs-rest patterns. The mov-vs-mov MI patterns are below the significance threshold (see Fig 11C). The mov-vs-rest MI patterns arise on central motor cortex areas at movement onset (see Fig 11D).

The time averaged ME patterns of mov-vs-mov and mov-vs-rest are similar and are located on PM, M1, S1 and PPC (see Fig 11E). The time averaged MI mov-vs-mov patterns are faintly located on central areas (see Fig 11G), whereas the mov-vs-rest patterns have a more distinct representation on M1 and S1.

## Discussion

We show in this work for the first time the successful classification of six different movements of the right arm from low-frequency time-domain EEG. Significant classification accuracies were reached during movement execution as well as during movement imagination. This proves that single and non-repetitive movements of the same limb can be decoded from time-domain EEG signals and differentiated against each other. However, despite the ME classification accuracies being promising, the MI classification accuracies are rather low. This may be because ME EEG signals were time-locked to the actual movement onset but MI EEG signals were time-locked to a virtual movement onset (which corresponded to the average ME onset of each subject). Thus, the ME onset was more accurate, and exact time-locking is important for classifying in the time-domain as the underlying signals change over time. One could overcome this issue by defining the virtual movement onset relative to occurring MRCPs [43] instead as a fixed time delay. Another explanation may be that ME produces more pronounced brain patterns than MI in the time-domain. This is indicated by studies analyzing MRCPs [43,44]. Interestingly, Sugata et al. did not find such a dissimilarity in classification accuracy between ME and MI in a magnetoencephalography (MEG) study using comparable features in grasping, pinching and elbow flexion [45]. Also Wang et al. obtained more comparable classification accuracies between ME and MI in a MEG based study employing a target decoding paradigm [46]. Beside that, attempted movements may produce more pronounced brain patterns than MI and therefore yield higher classification accuracies. They may cause a stronger activation of the motor system as indicated in Blokland et al. where classification accuracies in tetraplegic individuals were higher with attempted movements than MI using spectral features [47]. Furthermore, extensive user training could improve the expression of distinct brain patterns. User training can be highly beneficial in SMR-based BCIs [12,48], however it is still unclear if this is also true for time-domain signals in the context of movement decoding. Moreover, the obtained confusion matrices indicate that movements involving different joints (i.e. different muscle groups) are more discriminable than movements involving the same joints Consequently, for future applications it would be necessary to select the subset of classes which work best for BCI users but still allow a natural control. Furthermore, a hierarchical classifier concept may be beneficial: one meta classifier classifies movements of different joints (e.g. hand movement vs elbow movement), and subjacent classifiers classify movements of the same joint (e.g. hand open vs hand close).

A simple approach to improve the classification accuracy is to use more temporal information when classifying the EEG. Therefore, we also classified time windows instead of single time points of the EEG, and analysed the effect of the time window length. The results indicate that a time window of length 0.6 s is sufficient to reach the maximum possible classification accuracy (w.r.t. the methods used in this paper), longer time windows don't improve the classification performance and increase the computational load. Furthermore, ME classification profits more from a time window based approach than MI in case of mov-vs-mov. The improvement in classification performance can be due to the temporal spread of the discriminative information of the underlying signals (i.e. MRCPs) which is better captured with a time window based classification. Another reason may be that a time window based classification allows to fine-tune the employed 0.3–3 Hz bandpass filter. An LDA classifier which uses data from more than one time point is basically a finite impulse response filter with trainable filter coefficients, and can shrink or enlarge the 0.3–3 Hz passband to maximize the extracted discriminative information.

Earlier, we pointed out some possibilities to boost the MI accuracy. However, a study conducted by Lacourse et al. [49] forments doubts if MI accuracy in healthy subjects is a good predictor for the performance in SCI subjects. They found that MRCPs during attempted and imagined hand movements in tetraplegic subjects are more similar than in a abled-bodied control group (there with executed and imagined movements). Furthermore, they found that MRCPs between tetraplegic subjects and abled-bodied subjects are different. This challenges the usefulness of using MI in abled-bodied subjects to predict the classification performance for SCI subjects. Nevertheless, our results show the general applicability in able-bodied subjects and point out the need for further research in SCI subjects with attempted movements.

Our work adds to the work of Vučković and Sepulveda who have shown that wrist extension/flexion and forearm pronation/supination can be decoded from the frequency-domain of EEG [27,28] (especially from the delta band). Here, we show that also the time-domain contains movement information related to individual joint movements. This is in line with previous research which shows that low-frequency time-domain EEG signals contain information about movement trajectories, speed and force [17–19,22,23,26]. Electrocorticography (ECoG) studies support this and indicate that low-frequency time-domain signals contain movement related information [50–54]. Interestingly, the frequency bands used in classical SMR-based BCIs, i.e. mu and beta band, contain less information about movement kinematics and muscle activity than low-frequency bands and the high-gamma band [55–57]. Mu and beta bands are more suitable to detect a movement intention than the details of the movement. However, our group recently found that these frequency bands can be separated in two types of large-scale networks where one network type is modulated by the movement phase of rhythmic finger movements [9].

To reliably detect the movement intention is of utmost importance for a neuroprosthesis control to avoid unexpected and potentially dangerous movements. In accordance with [26,58], we successfully classified between movements and a rest class based on low-frequency time-domain EEG. The classification of movement vs rest may be further improved by combining time-domain signals and power modulations in mu and beta bands [59].

MRCPs can be retrieved with similar signal processing methods as low-frequency time-domain signals. They show a typical negative peak around movement onset like in our results [24]. Hence, our classification approach is based on MRCPs. Such MRCPs-like signals are also observable in both ME and MI rest classes, i.e. without any movement intention. It is reported that voluntary muscle relaxation causes similar potential changes to that of muscle contraction [24]. This may be an explanation at least for ME if the subjects were already preparing for some movement before the cue appeared on the screen, and then relaxed after the rest class cue was presented. This can be an issue for an asynchronous BCI trained with a cue based paradigm. An asynchronous BCI must be trained on a rest class which truly corresponds to a relaxation phase, and this requires a careful design of the training paradigm.

A novelty in the context of EEG-based movement decoding from a single limb is the analysis of the classifier patterns. These patterns show for ME that mainly M1, S1, PM, and PPC contain movement related information which can be decoded from low-frequency time-domain EEG signals. This is consistent with the general understanding that PM and PPC are involved in movement planning while M1 is active during the execution of the movement, and S1 receives proprioceptive feedback which is eventually integrated with other sensory input at the PPC [60–62]. The ME mov-vs-mov patterns show also a slight and temporary involvement of a non-motor related ipsilateral temporal area. However, this lateral pattern cannot be attributed to movement artefacts as the mov-vs-rest classifier would be more susceptible to movement artefacts but does not have similar pronounced lateral pattern. This lateral pattern can be a consequence of the usage of a template head model and an incomplete electrode coverage on temporal sites. Another observation is that mov-vs-mov and mov-vs-rest patterns cover similar areas. Thus, general (mov-vs-rest) and detailed (mov-vs-mov) movement information can be decoded from the same brain areas. One can also observe that MI produces less pronounced patterns than ME, which is consistent with a lower classification accuracy for MI than ME. The MI patterns are also more centrally located.

We calculated classifier patterns instead of analyzing the weights of the LDA classifier because the EEG channels are highly correlated in lower frequencies [19] which causes a problem known as multicollinearity [63] and complicates their interpretation [64]. Classifier patterns were already used as a tool to spatially analyze brain processes [65]. They can be used to find EEG amplitude differences exploited by the classifier between experimental conditions.

The following limitations of our study can be identified. First, preprocessing filter and classification time windows were non-causal to avoid time shifts in the obtained results due to signal processing. However, for an online application causal filter and time windows must implemented. Second, the movement onsets obtained via external sensors are not as timely as movement onsets obtained via electromyography. Due to inertia of the body parts, muscle activity is usually detected before overt movements. Third, we used template head models instead of individual head models generated from magnetic resonance imaging scans for source imaging, which can increase the location error of sources and in turn decreases the sensitivity of the obtained patterns.

Future studies need to confirm if details of imagined or attempted movements can also be decoded from individuals with SCI and if the classifier performance is sufficient to control a neuroprosthesis or a robotic arm. Specifically, it has to be determined if the classification accuracies yielded by attempted movements in individuals with SCI correspond closer to the ME or MI accuracies reported in this work. The classifier patterns show that PM, M1 and S1 encode information about the details of the movement on the macroscale, and especially these areas have direct connections to the spinal cord [62,66]. These direct connections are impaired in SCI users, however, and this could have an influence on the information encoded in the MRCPs [49]. Further studies also need to analyze the influence of object interactions on the movement information encoded in low-frequency time-domain EEG signals.

## Conclusion

We have demonstrated the successful decoding of single executed and imagined upper limb movements based on low-frequency time-domain EEG signals. These movements can be the basis for new mental control strategies aimed at a more natural neuroprosthesis or robotic arm control. Furthermore, we show that the patterns underlying the classification emerge from motor related brain areas.

## Supporting information

S1 Fig MRCPs for movements belonging to the same joints. Shown is the average over subjects.
(TIF)

S1 Video Progression of the ME mov-vs-mov patterns. Patterns were calculated for single time points (i.e. not averaged over time) from -1 s to 1 s relative to movement onset. Statistical analysis was not performed.
(AVI)

S2 Video Progression of the ME mov-vs-rest patterns. Patterns were calculated for single time points (i.e. not averaged over time) from -1 s to 1 s relative to movement onset. Statistical analysis was not performed.
(AVI)
