# Supplementary figures and images for: Upper limb movements can be decoded from the time-domain of low-frequency EEG
Source: PLoS One. 2017 Aug 10;12(8):e0182578. doi: 10.1371/journal.pone.0182578 (PMC5552335; doi:10.1371/journal.pone.0182578)

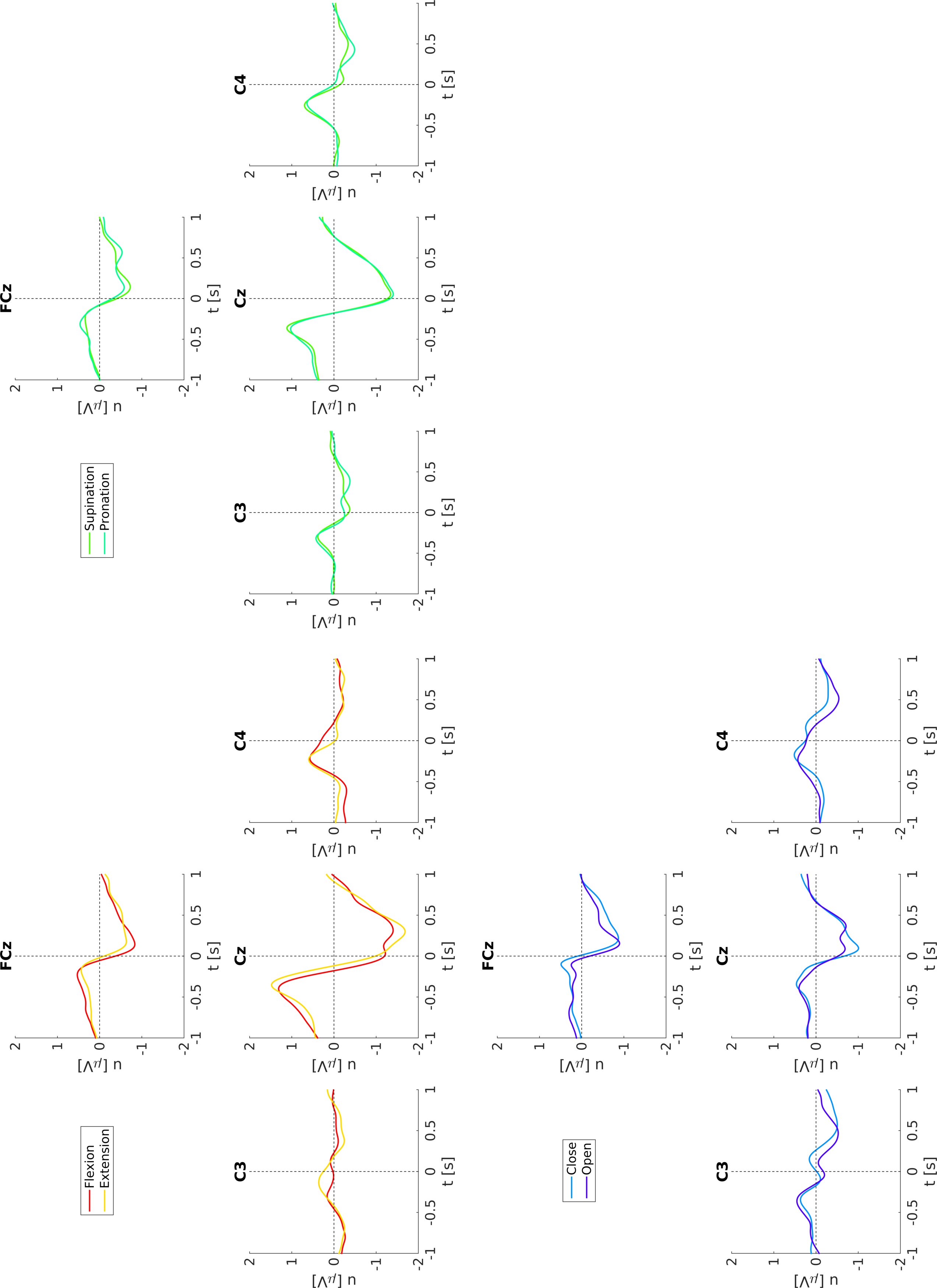

Supplement: S1 Fig — Shown is the average over subjects. (TIF) [file pone.0182578.s001.tif]
